# The complete mitochondrial genome and phylogenetic analysis of *Coscinodiscus wailesii* (Coscinodiscophyceae, Bacillariophyta)

**DOI:** 10.1080/23802359.2021.1934578

**Published:** 2021-06-03

**Authors:** Hailong Huang, Yichao Wang, Huiyin Song, Jing Wang, Yang Chen, Yongfang Zhao, Feng Liu, Nansheng Chen

**Affiliations:** a CAS Key Laboratory of Marine Ecology and Environmental Sciences, Institute of Oceanology, Chinese Academy of Sciences, Qingdao, China; b Laboratory of Marine Ecology and Environmental Science, Qingdao National Laboratory for Marine Science and Technology, Qingdao, China; c University of Chinese Academy of Sciences, Beijing, China; d Center for Ocean Mega-Science, Chinese Academy of Sciences, Qingdao, China; e Jiaozhou Bay National Marine Ecosystem Research Station, Institute of Oceanology, Chinese Academy of Sciences, Qingdao, China; f Department of Molecular Biology and Biochemistry, Simon Fraser University, Burnaby, British Columbia, Canada

**Keywords:** Diatoms, mitochondrial genome, *Coscinodiscus wailesii*, Coscinodiscophyceae

## Abstract

*Coscinodiscus* is a species-rich genus with about 400 species described, many of which are harmful algal bloom species with significant negative ecological impact. Despite of their importance in primary production and as harmful algal bloom species, genome data for species in this genus is limited. No mitochondrial genome (mtDNA) of any species in this genus has been reported. Here, the complete mtDNA sequence of the *Coscinodiscus wailesii* Gran & Angst 1931 was constructed and analyzed. The circular mtDNA was 36,071 bp in length, encoding 64 genes, including 34 protein coding genes (PCGs), 24 transfer RNA (tRNA) genes, 2 ribosomal RNA (rRNA) genes and 4 conserved open reading frames (*orf*s). The overall AT content of *C. wailesii* mtDNA was 75.00%, which was slightly lower than that of *Melosira undulate* (78.40%). Maximum likelihood (ML) phylogenetic analysis using 29 shared protein-coding genes revealed that *C. wailesii* clustered well with *M. undulata*, which was the only species of class Coscinodiscophyceae whose mtDNA has been fully constructed. The complete mtDNAs of more *Coscinodiscus* species will be valuable for studying the evolutionary relationships among species in the genus *Coscinodiscus* and in the Class of Coscinodiscophyceae.

Diatoms are a diverse phytoplankton group estimated to contribute up to 20% of global primary production (Mann 1999). *Coscinodiscus wailesii* belongs to the genus *Coscinodiscus*, the class Coscinodiscophyceae, and the phylum Bacillariophyta with a worldwide distribution (Rick and Dürselen 1995). The genus *Coscinodiscus* is species-rich with about 400 species identified globally (Li 2009) and 49 species identified in China (Xie 2006), many of which have been found to form blooms with severe negative impact directly on environment (Hasle and Lange 1992). The cell size of *Coscinodiscus* species is large, about 70–470 µm, with a high carbon content, which poses a great impact on the whole carbon pool and plays an important role in the marine ecosystem (Sun and Liu 2005). *Coscinodiscus wailesii* is a harmful algal bloom (HAB) species that can cause serious damage to Nori (*Pyropia*) cultivation through competitive utilization of nutrients during its bloom (Tetsuya et al. 2000; Nishikawa et al. 2010), and forms dense HABs that cause extensive clogging of fishing nets and other equipment, such as cages (Laing and Gollasch 2002).

Mitochondrial genome is an ideal handle for genetic and phylogenetic analyses because of its uniparental inheritance and high evolutionary rates (Wang et al., 2016). Although the mtDNAs of many species in phylum Bacillariophyta have been sequenced, most of these mtDNAs were constructed for species of two of the three classes including Mediophyceae and Bacillariophyceae. By now, mtDNA of only a single species (e.g., *M. undulata*) in the third class Coscinodiscophyceae has been constructed. The first aim of this study was to construct mtDNA of the spcies *C. wailesii*, which represents the second mtDNA of species of the class Coscinodiscophyceae. The second aim was to compare the two mtDNAs of these two species *C. wailesii* and *M. undulate* in the class Coscinodiscophyceae. Here, we assemble and annotate the complete mtDNA of *C. wailesii* for the first time, as part of our effort to gain better understanding of its genetic characteristics at the genome level.

The strain *C. wailesii* CNS00372 used in this study was isolated in water samples collected during an expedition to the Jiaozhou Bay, China (36°08.364′N, 120°11.359′E) in September 2019 onboard the research vehicle ‘Innovation’. Its specimen was deposited in the collection of marine algae in KLMEES of IOCAS (Nansheng Chen, chenn@qdio.ac.cn) under the voucher number CNS00372. The annotation of protein-coding genes (PCGs), transfer RNA (tRNA) genes, ribosomal RNA (rRNA) genes and open reading frames (*orf*s) were conducted using Open Reading Frame Finder (ORF finder) (https://www.ncbi.nlm.nih.gov/orffinder), tRNAscan-SE 2.0 (Chan and Lowe 2019) and MFannot (https://megasun.bch.umontreal.ca/RNAweasel).

The circular mtDNA of *C. wailesii* was 36,071 bp in size (GenBank accession number: MW122841). The mtDNA encoded a set of 64 genes found in most diatom mtDNAs (Wang et al. 2021; Zhang et al. 2021), including 34 protein-coding genes (PCGs), 2 ribosomal RNA (rRNA) genes, 24 transfer RNA (tRNA) genes, and 4 open reading frames (*orf*s). The heavy strand (+) encoded 56 genes while the light strand (–) had 8 genes. The percentage of A + T of the mtDNA of *C. wailesii* was 75.00%, which was slightly lower than that of *M. undulata*. The 34 PCGs included *atp6*,*8*,*9*; *cob*; *cox1-3*; *nad1-7*,*4L*,*9*,*11*; *rpl2*,*5*,*6*,*14*,*16*; *rps3*,*4*,*7*,*8*,*10-14*,*19*; and *tatA*,*C*. All PCGs began with ATG except *atp8*, which began with TTA. The termination codons of PCGs were either TAA (29 of 34 genes) or TAG (5 of 34 genes). The 24 transfer RNA (tRNA) genes ranged in size from 71 bp to 89 bp. Five pairs of overlapping genes were identified in the *C. wailesii* mtDNA, including *rps10*-*trnF* (10 bp), *rps12*-*rps7* (65 bp), *nad1*-*tatC* (48 bp), *orf143*-*trnP* (9 bp), and *rrs*-*trnS* (53 bp).

Maximum likelihood (ML) phylogenetic tree was constructed using concatenated amino acid sequences of 29 protein-coding genes (*atp 8*, *9*; *cob*; *cox1*, *2*, *3*; *nad1-7*, *4 L*, *9*; *rpl2*, *5*, *6*, *14*, *16*; *rps3*, *4*, *8*, *10*, *11*, *13*, *14*, *19*; and *tatC*) shared by 33 publicly available diatom mtDNAs (Figure 1), using IQtree v1.6.12 (Trifinopoulos et al. 2016) with 1000 bootstrap replications. Two species *Sargassum fusiforme* (KJ946428) and *Sargassum muticum* (KJ938301) of Ochrophyta were used as out-group taxa. The results demonstrated that species fell well into three clades corresponding to three classes of the phylum Bacillariophyta, including Coscinodiscophyceae, Mediophyceae and Bacillariophyceae. In a separate study, Crowell et al. (2019) showed that *Durinskia baltica* and the two *Nitzschia palea* plastomes grouped on a strongly supported branch (bootstrap 100) within a larger clade that included *Kryptoperidinium foliaceum*. The phylogenetic positions of Endosymbiont of *K. foliaceum* and *D. baltica* are consistent with our results described in this manuscript. Recent studies have also proposed that the species *Thalassiosira pseudonana* belongs to the genus *Cyclotella* (Alverson et al. 2011). As expected, mtDNA of *C. wailesii* (MW122841) showed a close relationship with that of *M. undulata* (NC037728) (Figure 1), which was the only species in the class Coscinodiscophyceae whose mtDNA had been fully constructed (Pogoda et al. 2019). Comparative analysis of mtDNAs of *C. wailesii* and *M. undulata* revealed extensive genome rearrangement events including translocation and inversion events. Nevertheless, many conserved gene blocks could be identified, such as *atp6*-*rps10*-*trnF*(gaa)-*rps8*-*rpl6*. These results suggested that mtDNAs of many more species of the class Coscinodiscophyceae are needed for in depth understanding of their evolutionary dynamics.

**Figure 1.**
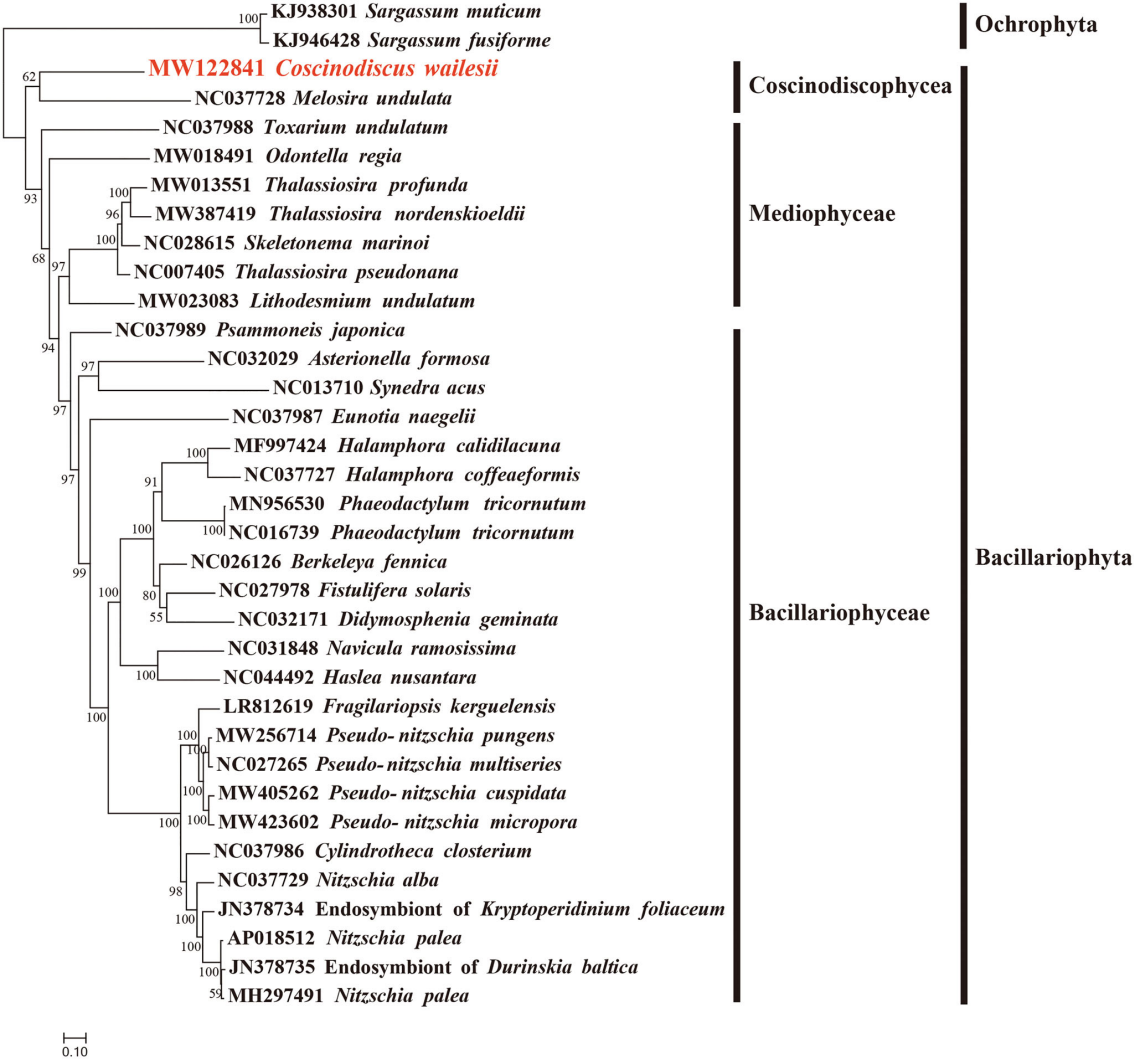
Maximum likelihood phylogenetic tree using concatenated amino acid sequences of 29 shared protein-coding genes (*atp 8*, *9*; *cob*; *cox1*, *2*, *3*; *nad1-7*, *4 L*, *9*; *rpl2*, *5*, *6*, *14*, *16*; *rps3*, *4*, *8*, *10*, *11*, *13*, *14*, *19*; and *tatC*) from 33 publicly available diatom mtDNAs, and *Sargassum fusiforme* (KJ946428) and *Sargassum muticum* (KJ938301) of Ochrophyta were used as out-group taxa. The numbers beside branch nodes are the percentage of 1000 bootstrap values.

## Data Availability

The genome sequence data that support the findings of this study are openly available in GenBank of NCBI at https://www.ncbi.nlm.nih.gov/under the accession number MW122841. The associated BioProject, SRA and Bio-Sample numbers are PRJNA686182, SRR13269809 and SAMN17108303, respectively.

## References

[CIT0001] Alverson AJ, Beszteri B, Julius ML, Theirot EC. 2011. The model marine diatom *Thalassiosira pseudonana* likely descended from a freshwater ancestor in the genus *Cyclotella*. BMC Ecol Evol. 11:125–125. 10.1186/1471-2148-11-125 PMC3121624 21569560

[CIT0002] Chan PP, Lowe TM. 2019. tRNAscan-SE: searching for tRNA genes in genomic sequences. Methods Mol Biol. 1962:1–14. 31020551 10.1007/978-1-4939-9173-0_1 PMC6768409

[CIT0003] Crowell RM ,Nienow JA ,Cahoon AB. 2018. The complete chloroplast and mitochondrial genomes of the diatom *Nitzschia palea* (Bacillariophyceae) demonstrate high sequence similarity to the endosymbiont organelles of the dinotom *Durinskia baltica*. J Phycol. 55(2):352–364. 10.1111/jpy.12824 30536677

[CIT0004] Hasle GR, Lange CB. 1992. Morphology and distribution of *Coscinodiscus* species from the Oslofjord, Norway, and the Skagerrak. North Atlantic Diatom Res. 7(1):37–68.

[CIT0005] Laing I, Gollasch S. 2002. Coscinodiscus wailesii – a nuisance diatom in European waters. Invasive aquatic species of Europe. Distribution, impacts and management. Amsterdam (The Netherlands): Springer.

[CIT0006] Li Y. 2009. Morphological characteristics comparisons between *Thalassiosira* and *Coscinodiscus*. Bull Bot Res. 29(3):282–288.

[CIT0007] Mann DG. 1999. The species concept in diatoms. Phycologia. 38(6):437–495.

[CIT0008] Nishikawa T, Tarutani R, Yamamoto R. 2010. Nitrate and phosphate uptake kinetics of the harmful diatom *coscinodiscus wailesii*, a causative organism in the bleaching of aquacultured *porphyra thalli*. Harmful Algae. 9(6):563–567.

[CIT0009] Pogoda CS, Keepers KG, Hamsher SE, et al. 2019. Comparative analysis of the mtDNAs of six newly sequenced diatoms reveals group II introns in the barcoding region of cox1. Mitochondrial DNA Part A. 30(1):43–51. 10.1080/24701394.2018.1450397 29527965

[CIT0010] Rick HJ, Dürselen CD. 1995. Importance and abundance of the recently established species *Coscinodiscus wailesii* Gran & Angst in the German Bight. Helgol Mar Res. 49(1):355–374.

[CIT0011] Sun J, Liu D. 2005. Net-phytoplankton community of the Bohai Sea in the autumn of 2000. Acta Oceanolog Sin. 27(3):124–132.

[CIT0012] Tetsuya N, Kazutaka M, Satoshi N. 2000. Effects of temperature and salinity on the growth of the giant diatom *Coscinodiscus wailesii* isolated from Harima-Nada, Seto Inland Sea, Japan. Nippon Suisan Gakkaishi. 66(6):993–998.

[CIT0013] Trifinopoulos J, Nguyen LT, von Haeseler A, Minh BQ. 2016. W-IQ-TREE: a fast online phylogenetic tool for maximum likelihood analysis. Nucleic Acids Res. 44(W1):W232–W235. 27084950 10.1093/nar/gkw256 PMC4987875

[CIT0014] Wang S, Fan X, Guan Z, Xu D, Zhang X, Wang D, Miao Y, Ye N. 2016. Sequencing of complete mitochondrial genome of *Saccharina latissima* ye-C14. Mitochondrial DNA Part A. 27(6):4037–4038. 10.3109/19401736.2014.1003832 25629480

[CIT0015] Wang Y, Chen Y, Wang J, Liu F, Chen N. 2021. Mitochondrial genome of the harmful algal bloom species *Odontella regia* (Mediophyceae, Bacillariophyta). J Appl Phycol. 33(2):855–868.

[CIT0016] Xie W. 2006. Community structure and dynamics of planktonic diatoms in typical areas of East China Sea. Xiamen University, Xiamen, China.

[CIT0017] Zhang M, Cui Z, Liu F, Chen N. 2021. Definition of a high-resolution molecular marker for tracking the genetic diversity of the harmful algal species *Eucampia zodiacus* through comparative analysis of mitochondrial genomes. Front Microbiol. 12.. 10.3389/fmicb.2021.631144 PMC8024477 33841358

